# Study of the Cutting Performance of Ti-6Al-4 V Alloys with Tools Fabricated with Different Microgroove Parameters

**DOI:** 10.3390/ma18184312

**Published:** 2025-09-15

**Authors:** Liang Xu, Dayong Yang, Zhiyang Zhang, Min Liu

**Affiliations:** 1 School of Mechanical and Automotive Engineering, Guangxi University of Science and Technology, Liuzhou 545006, China; 2 College of Intelligent Manufacturing, Jingchu University of Technology, Jingmen 448000, China

**Keywords:** microtextured cutting tools, microgroove parameters, cutting performance of cutting tools, laser-processed texture, titanium alloy (Ti-6Al4 V)

## Abstract

Microtextured cutting tools are widely used because of their excellent performance in cutting difficult-to-machine materials. The cutting performance of cutting tools largely depends on the size parameters of the microtextures used. This study focuses on the machining of titanium alloy Ti-6Al4 V using microgrooved cutting tools under dry-cutting conditions. Special emphasis is placed on exploring cutting performance under specific combinations of microgroove parameters. To determine the optimal parameter combination for cutting, the effects of different microgroove parameters (including the diameter, depth, spacing, and spacing between grooves and cutting edges) on cutting force, tool wear, and chip morphology were investigated. In this study, femtosecond laser technology was used to prepare microgroove-textured cutting tools with different parameters, and the cutting performance of these tools was analyzed. The results show that, when the groove diameter is 80 μm, the depth is 60 μm, the spacing is 80 μm, and the distance between the groove and the tool tip is 120 μm, the cutting performance of the tool is optimal: the cutting force is reduced by 13.9%, the degree of tool wear is minimized, and the degree of chip curling is more uniform. The research results can be applied to the actual processing of Ti-6Al4 V, which can help tool design, selection, and microtexture parameter optimization.

## 1. Introduction

Cutting is a crucial process and plays a pivotal role in the manufacturing industry [1]. It is used for manufacturing numerous core components, and is widely used in multiple key industries [2,3]. This is especially true in key fields such as energy, chemical engineering, and aerospace [4,5]. To improve the cutting efficiency of cutting tools, researchers have taken various measures, such as developing new tool materials, applying surface microtextures, optimizing tool geometry, and using coating treatments [6]. Among these measures, the potential of surface microtexture technology is particularly promising [7,8]. The microtextured tool completely abandons the use of cutting fluid in the dry-cutting process, cuts off the emission path of such pollutants from the source, effectively avoids the long-term cumulative harm of harmful substances to the environmental medium, and significantly reduces the energy consumption and reagent consumption of industrial wastewater treatment. Notably, this technology is in line with the concepts of green processing and green development, aligning with environmental protection and sustainable development objectives in modern manufacturing [9]. Compared to nontextured surfaces, textured surfaces have significant advantages in terms of antiadhesion, drag reduction, antifouling, anticorrosion, and self-cleaning [10,11].

In recent years, numerous scholars have proposed and confirmed that machining microtextures with different morphological features, orientations, and composite morphologies on tool surfaces can significantly increase the cutting function of tools during the machining process. In terms of texture morphology, Zheng K et al. [12] experimentally studied the performance of microtextured tools when cutting Ti-6Al-4V. They studied three kinds of textures on the front cutting surface: linear grooves, diamond grooves, and sinusoidal grooves. Their findings reveal that sine-textured tools excel in terms of cutting performance, followed by line-textured tools, whereas diamond-textured tools have relatively poor performance. Texture-enhanced cutting tools can decrease the primary cutting force by as much as 30.97%. Palanivel R et al. [13] created four texture patterns on the front blade surface: concentric circles, squares, crosses, and diagonals. Their research reveals that diagonal-patterned cutting tools have the best machining characteristics, with a cutting force reduction of 34% and a surface smoothness improvement of 37% at 150 m/min. In terms of textures in different directions, Sun X et al. [14] machined textures in three different directions on the front cutting surface of hard alloy tool heads: vertical, parallel, and perpendicular to the main cutting edge. Their research reveals that parallel-textured tools can decrease the friction coefficient by 14%. The wear on the front cutting edge of the tool decreased by 35–70%, whereas surface roughness was enhanced by 29%. Arulkirubakaran et al. [15] created various texture patterns, such as regions and linear, parallel, and vertical grooves, on the front cutting surface of a tool to investigate the effects on Al Cu/TiB turning. Their results show that using tools perpendicular to the cutting edge texture reduced the cutting force by 18% and that the cutting temperature significantly decreased by 16–22%. In terms of the microstructure of composite morphology, Wu F et al. [16] researched the tool wear performance of PCD tools with pits, grooves, and mixed microstructures when cutting SiCp/Al composites in terms of the microstructure of composite morphology. Their results show that the presence of mixed microtextures can effectively control tool wear and extend tool life. Wear of the mixed-microtextured cutting tool decreased by 53.8%, indicating optimal wear reduction performance. Yu Z et al. designed a texture composed of micropits and microgrooves using laser processing to investigate the effect on the cutting performance of tungsten alloys. Their results show that when composite-textured cutting tools are used, cutting force can be reduced by up to 9%, and the maximum reduction in surface roughness can be up to 39.3% [17]. In addition, in terms of microtexture parameters, Yang et al. [18] used laser processing technology in a dry-cutting environment, and used milling force as an evaluation index to obtain the following optimal parameters: the microtexture diameter of the rake face is 50 μm, the correlation distance with the texture is 200 μm, and the distance from the cutting edge is 110 μm. Hu et al. [19] used a femtosecond laser to prepare micro-pit textures with different parameters. By analyzing the tool wear, the optimal anti-wear parameters were determined: the micro-pit was 60 μm away from the tool, the diameter was 70 μm, and the spacing was 100 μm. Gong et al. [20] optimized the microtexture dimensions (diameter, depth, and distance) of laser processing, and obtained the optimal values of 126, 15 and 200 μm, respectively.

Currently, the hotspots in research are focused mostly on the morphology of microtextures, but research on the surface sizes of microtextures is lacking. In view of this, this article focuses on exploring the influence of surface microtexture size on tool cutting performance. Femtosecond laser processing utilizes the interaction between an ultrashort-pulse laser and materials to achieve high-precision and low-thermal-damage micromachining. With its unique advantages, femtosecond laser processing has become one of the core processes in the field of micro–nano manufacturing. Based on this, this paper selects femtosecond laser processing technology to process tools. Using cutting experiments, the cutting performance of microgrooved cutting tools with various parameter combinations was analyzed, with a focus on exploring the effects of differences in microgroove parameters on the cutting force, degree of wear, and chip morphology during titanium alloy machining.

## 2. Experiment

### 2.1. Microtexture Fabrication

Among the various surface-texture preparation techniques, laser processing has outstanding advantages, with a high material removal rate [21], good controllability [22], the ability to reduce substrate surface contamination [23], and the ability to handle complex surface-texture preparation scenarios [24]. It involves noncontact machining [25], which avoids problems related to tool deformation caused by mechanical stress and can keep the substrate intact. Moreover, laser processing is applicable to various tool materials, and its advantages are fully revealed when working with difficult-to-machine materials [26].

**Figure 1:**
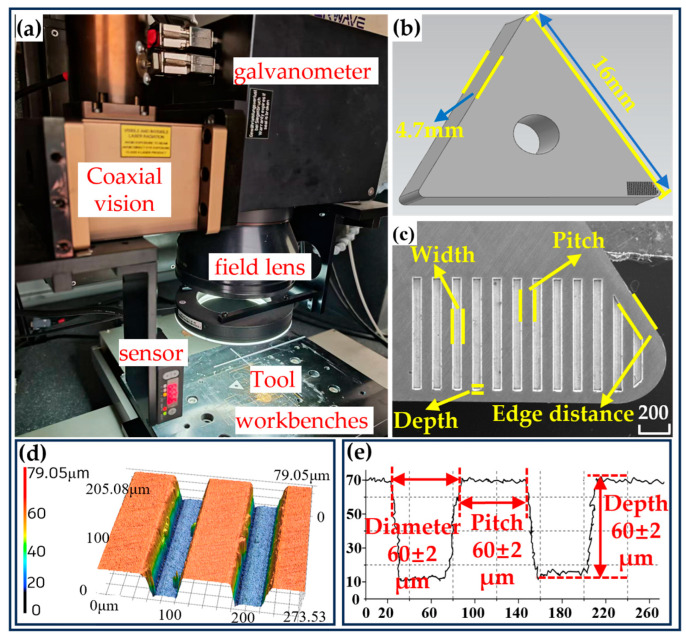
Laser processing equipment and microstructure morphology. (**a**) Femtosecond laser processing equipment. (**b**) Appearance of tool texture. (**c**) Microstructure SEM image. (**d**) Three-dimensional texture morphology. (**e**) Three-dimensional texture size parameters.

Therefore, in this study, microtextured grooves were prepared on the surface of a tool with dimensions of 16 mm × 16 mm × 4.7 mm and a tool tip radius of 0.4 mm using the femtosecond laser processing method. The processing machine was an LR-fmto515-20 (Nanjing Shengzi Guangxun Intelligent Technology Co., Ltd., Nanjing, China), as shown in Figure 1a. The processing conditions were as follows: repetition rate of 40 kHz, energy of 30 μJ, and spot movement speed of 0.5 mm/s. The appearance of the cutting tool is depicted in Figure 1b. The distribution and size of the grooves on cutting tools are extremely important, as the texture properties can be fully utilized during the cutting process only through a reasonable combining of texture parameters. To achieve this, four texture parameters—the diameter, depth, spacing, and edge distance of the cutting tool—were chosen, employing a Taguchi L9 orthogonal design (with four factors at three levels) [27,28,29]. The geometric shape and distribution of the textures are shown in detail in Figure 1c. With the help of a Keyence laser confocal microscope VK-X150 (Keyence, Itasca, IL, USA), testing was carried out, and three-dimensional morphological images, as shown in Figure 1d, corresponding to the different processing parameters, were successfully obtained. Moreover, the three-dimensional morphological size of the laser-processed texture was measured, as shown in Figure 1e.

### 2.2. Experimental Equipment

The Ti-6Al-4V titanium alloy, renowned for its high strength-to-weight ratio, exceptional corrosion resistance, and favorable biocompatibility, is extensively utilized in the aerospace, automotive, and medical industries [30,31]. However, the modern manufacturing industry has increasingly stringent requirements for the surface quality and machining accuracy of titanium alloy parts [32]. The low thermal conductivity and high strength of the Ti-6Al-4V alloy, as well as its interactions with cutting tools, greatly increase its difficulty in processing, making it prone to processing performance and surface quality issues [33,34]. Based on this, this titanium alloy was selected as the workpiece material in this study, as studying its machining performance is highly important.

**Figure 2:**
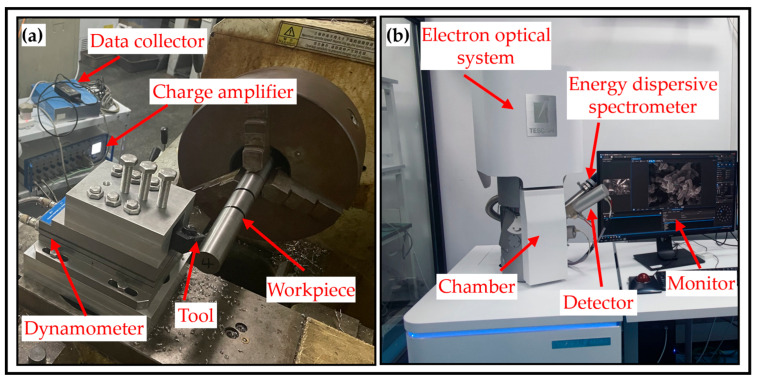
Microtextured tool cutting and testing device. (**a**) Microtextured tool cutting device. (**b**) Testing device.

In this study, turning experiments were conducted on the Ti-6Al-4V titanium alloy using microgroove-textured cutting tools in a dry-cutting environment. A C6140A lathe produced by Guangzhou Machinery Machine Tool Group Co., Ltd. in Guangzhou, China, was used to process Ti-6Al-4V titanium alloy rods with a size of Φ 30 × 300 mm. The cutting tool was made of a hard alloy produced by Tungaloy Co., Ltd. in Fukushima, Japan, model TNMA160404TH03, with a tool size of 16 mm × 16 mm × 4.7 mm, a tool tip radius of 0.4 mm, and a CWB-MTJNR1616H16 tool holder (KOESOTR, Taizhou, China). In the experiments, a model 9441 instrument produced by Kistler Instrument AG (Winterthur, Switzerland) was used in conjunction with a charge amplification device (model 5019A) to convert electrical signals into force signals. The experimental data were then collected and analyzed using a data acquisition device (model DA, 3605A) and supporting analysis software (Dynoware 4.1). As shown in Figure 2a. All of the experimental groups had consistent cutting parameters: a spindle speed of n = 1120 rpm, a feed rate of f = 0.2 mm/r, and a cutting depth of ap = 0.3 mm. Moreover, measurements and analyses were conducted using scanning electron microscopy (SEM model Czech TESCAN MIRA LMS (TESCAN, Brno, Czech Republic)) and energy dispersive spectroscopy (EDS model Oxford Xplore 30 (Oxford Instruments, Oxford, England)), as shown in Figure 2b.

### 2.3. Experimental Methods

In traditional experimental designs, increasing the number of process parameters, level, and number of repetitions can make an experiment complex and lengthy. The Taguchi method was developed for this purpose; this method can efficiently lock the optimal parameter combination, greatly reduce the number of experiments, and provide a robust and optimized systematic solution for tool design. Therefore, in this study, a Taguchi L9 orthogonal design (with four factors and three levels) was adopted to evaluate the influence of texture parameters on tool performance. Nine different combinations of texture parameters were set. The parameter levels selected for the experiment are detailed in Table 1. Table 2 presents the nine texture parameter combinations obtained through texture processing on the experimental tool samples. Among them, the tools without texture processing are named NT, and the microgrooved tools with different texture parameters are named T1–T9 in sequence.

When evaluating cutting tool performance, cutting force is an important parameter that can be directly measured and evaluated. In this study, the signal-to-noise ratio (*S*/*N*) of the cutting force was calculated. The signal represents the expected value of the output response—that is, the average value; noise represents the standard deviation, which is an unexpected value in the output response. The cutting force *S/N* ratio was calculated using Equation (1) [35,36]. Regardless of the combination of texture parameters, the higher the signal-to-noise ratio, the better the tool performance and the more significant the effect on the signal-to-noise ratio rendering.

(1) $$\frac{S}{N}=-10*\log\left(\frac{\sum Y^{2}}{n}\right)$$

where *Y* is the measured data and *n* is the quantity of data.

On the basis of the analysis of variance (ANOVA) results of the orthogonal experimental design, a systematic analysis of the effects of the microgroove parameters was conducted to determine the importance ranking of the texture parameters.

The wear of the cutting tools during cutting can directly reflect their cutting performance. Electron microscopy (SEM) can be used to observe the microstructure, structural characteristics, and wear distribution of tool wear, and energy dispersive spectroscopy (EDS) can be used to investigate changes in composition, element migration phenomena, and chemical reactions during the wear process. In this study, scanning electron microscopy (SEM) and energy dispersive spectroscopy (EDS) were used to measure and analyze the wear surface of cutting tools from two perspectives: morphological characteristics and composition.

Chip curl, a key characteristic of chip morphology in the cutting process, has a direct and close relationship between its numerical value (usually characterized by the curl radius) and the cutting capability of the tool (including cutting force and tool wear). On this basis, in this study, a comparative analysis of chips generated using microgrooved cutting tools with different parameter combinations was conducted.

## 3. Results and Discussion

### 3.1. Cutting Force of Nontextured and Textured Tools with Different Parameters

**Figure 3:**
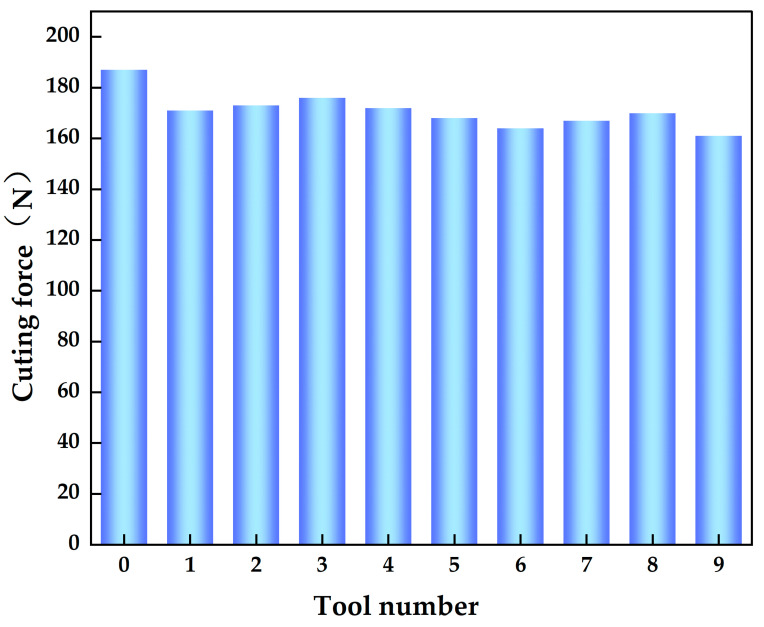
Cutting force of nontextured and textured tools with different texture characteristics.

The experimental results for the cutting force of nontextured tools and nine different groove-textured tools for a dry-cut titanium alloy with a spindle speed of 1120 rpm, a feed rate of 0.2 mm/r, and a cutting depth of 0.3 mm are shown in Figure 3. The experimental results indicate that, under the same cutting conditions, the measured cutting force for the nine different groove-textured tools were lower than those of the untextured tools, with a reduction range of 6.5–13.9%. Compared to the other tools, the T9 tool with a diameter of 80 μm, depth of 60 μm, spacing of 80 μm, and distance of 120 μm had the greatest reduction in cutting force, reaching 13.9%.

**Figure 4:**
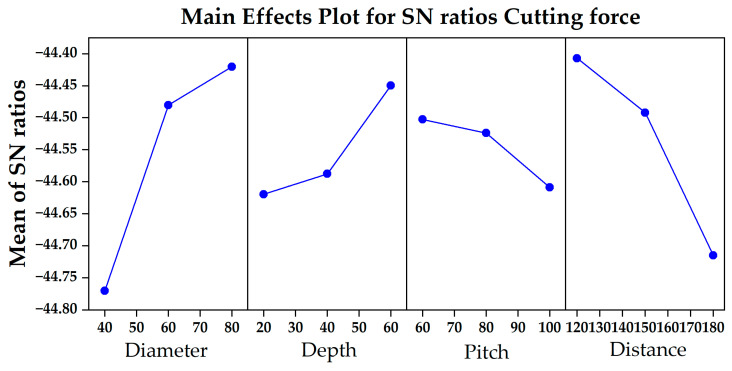
Influence of texture parameters on cutting force.

The impact of texture parameters on cutting force is shown in Figure 4. An analysis of the signal-to-noise ratio reveals that the higher the signal-to-noise ratio, the better the tool performance, and the better the performance on the signal-to-noise ratio effect map. Notably, the cutting force decreases as the texture unit diameter and depth increase, but increases as the spacing and edge distance increase. The influence of texture diameter on cutting force is the greatest, followed by edge distance, depth, and spacing. Increasing the texture diameter or minimizing its distance from the cutting edge (ensuring that the cutting edge strength is not weakened) notably reduces the cutting force. The effect is not significant when the texture depth varies between 20 μm and 40 μm, but it increases when the depth reaches 60 μm. In contrast, texture spacing has the smallest effect; when the texture unit is in the range of 60–80 μm, the texture is effective, but increasing the spacing further weakens this effect. From this, it can be determined that the minimum cutting force is obtained when the texture parameters are a diameter of 80 μm, a depth of 60 μm, a spacing of 80 μm, and a distance of 120 μm from the cutting edge. Table 3 presents the results of the analysis of variance (ANOVA) conducted on the texture parameters considered for the cutting force. The results of the F test reveal that, from the perspective of percentage contribution, the diameter of the texture unit is a key parameter, with a contribution rate close to 50%; the distance and depth between the texture and cutting edge follow; and the contribution of the spacing parameter is the smallest, being approximately 4%.

There is close consistency between the experimental results from different texture parameter combinations, the signal-to-noise ratios calculated on the basis of these test results, and the results obtained in the variance analysis. Moreover, the results of the signal-to-noise ratio analysis and variance analysis effectively validate the experimental results from different perspectives.

### 3.2. Tool Wear of Nontextured and Textured Tools with Different Texture Parameters

#### 3.2.1. Tool Rake Face Wear

**Figure 5:**
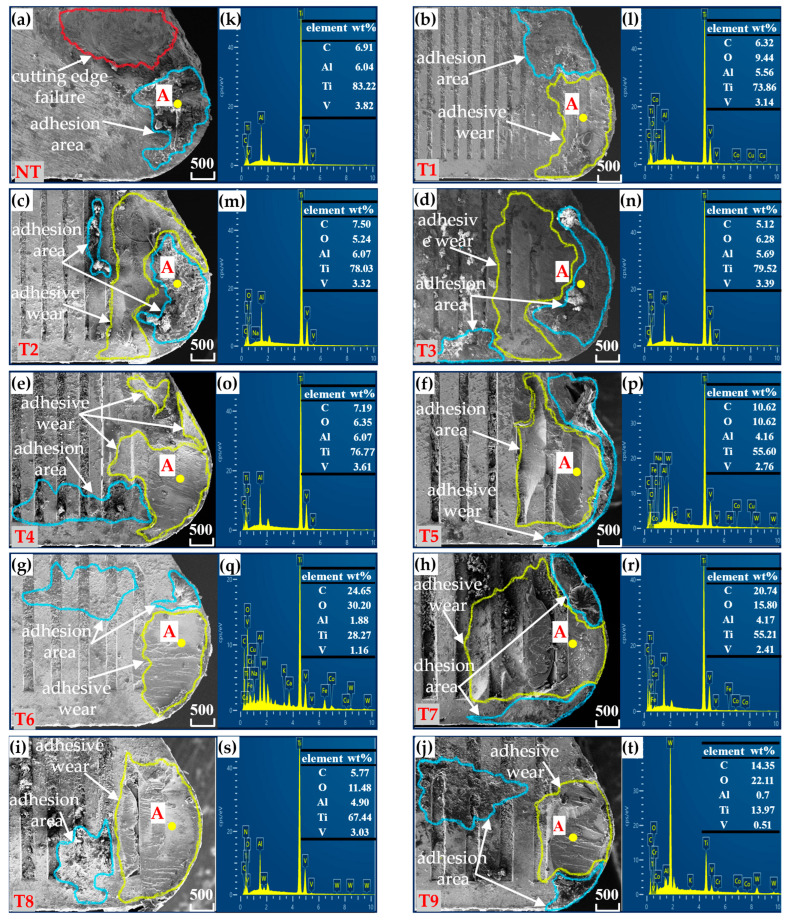
SEM and EDS images of the rake face of tools without textures and tools with different microtextures. (**a**–**j**): SEM wear morphology of the rake face ((**a**): Non textured cutting tools (NT); (**b**–**j**): Micro textured cutting tools with different parameter combinations (T1–T9)), (**k**–**t**) EDS elemental analysis corresponding to point “A” in (**a**–**j**).

To compare the differences in wear between tools without microtextures and those with microtextures of different characteristics, scanning electron microscopy (SEM) was used to observe the surface morphology of the front cutting surface of the tools. The wear morphology of the front cutting surface of nonmicrotextured tools during Ti-6Al4 V machining is shown in Figure 5a, whereas that of tools with grooved microtextures with different parameters is shown in Figure 5b–j, which displays SEM images of the tool’s surface morphology. During tool turning, significant wear occurs on the cutting face. For nontextured cutting tools, the front cutting surface shows pitting wear, as shown in Figure 5a. This type of pitting wear weakens the strength of the cutting edge, causing damage to the cutting edge. In contrast, Figure 5b–j shows the wear morphology of the front cutting surface of the microtextured tools under different parameter combinations. There are varying degrees of adhesive wear and adhesive areas on the front cutting surface of these tools, but none of them show the unique pitting wear of the nontextured tools, and the wear pattern is markedly superior to that of the nontextured tools. Among the tools, the T9 tool front face in Figure 5j has the most favorable degree of adhesion wear and adhesion area state.

Figure 5k–t shows the EDS energy spectrum analysis, focusing on the elemental composition of point A near the front cutting edge (the position of point A is shown in Figure 5a–j). The analysis results indicate that region A contains a high proportion of Ti and a small amount of Al, and C, W, O, and V are also detected. Given that the workpiece is rich in titanium (Ti), it can be inferred that the workpiece material adheres in the vicinity of the tool’s tip. By comparing the titanium content in area A, the wear condition of the cutting tool can be evaluated. Among the tools, the titanium content in area A of the T9 tool in Figure 5t is the lowest, indicating that its degree of wear is the lowest. The surface morphology of the front cutting surface and the results of the EDS energy spectrum analysis directly reflect the rationality of parameter selection and are consistent with the cutting force test results.

#### 3.2.2. Tool Flank Wear

**Figure 6:**
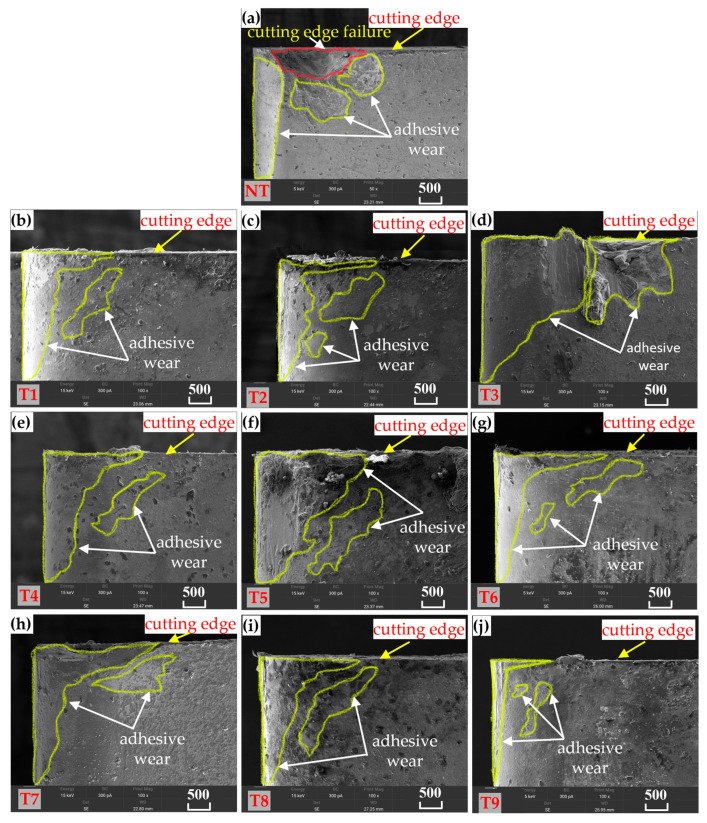
SEM images of flank wear for tools with no textures and tools with different microtextures (**a**): Non textured cutting tools (NT); (**b**–**j**): Micro textured cutting tools with different parameter combinations (T1–T9).

Similarly, scanning electron microscopy (SEM) was used to examine the facial morphology of the back cutting surface of the tool. The wear morphology of the rear cutting surface when Ti-6Al-4V was machined with nonmicrotextured tools is shown in Figure 6a, whereas the wear condition of the rear cutting surface when this material was machined with groove microtextured tools with different parameters is shown in Figure 6b–j, and the wear condition is approximately the same as that of the front cutting surface. The microtexture on the back cutting surface can minimize the area of contact with the machined surface of the workpiece, thereby delaying peeling on the back cutting surface of the tool. For nontextured cutting tools, the back cutting surface exhibits pitting wear, as shown in Figure 6a. This type of pitting wear reduces the strength of the cutting edge, ultimately leading to cutting edge damage. In contrast, Figure 6b–j presents the wear condition of the back cutting surface of the microtextured cutting tools with different parameter combinations. These cutting tools have varying degrees of adhesive wear and an adhesive area on the back cutting surface, but none of them show the unique pitting wear of the nontextured cutting tools, and the overall degree of wear is significantly lower than that of the nontextured cutting tools. Among the tools, the T9 tool in Figure 6j has the most ideal degree of adhesion wear and condition of adhesion area, which indicates an appropriate parameter combination and is consistent with the cutting force test results.

### 3.3. Analysis of the Chip Morphology of Tools with No Texture and Tools with Different Textures

**Figure 7:**
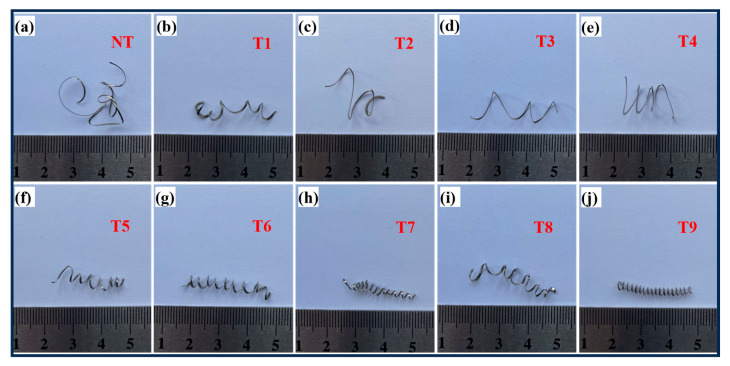
Chip images for tools without textures and tools with different microtextures (**a**): chip morphology of non textured cutting tools (NT); (**b**–**j**): Chip morphology of cutting tools with different parameter combinations (T1–T9).

As shown in Figure 7a–j, the degree of chip curling resulting from ordinary cutting tools and microtextured cutting tools with different parameters varies during cutting. The curling radius of chips generated using ordinary cutting tools (NTs) is greater than that of chips processed using textured cutting tools. When cutting with ordinary cutting tools, the length of contact between the chips and the front cutting surface is large, the friction area is large and unstable, the cutting force fluctuates slowly with contact, and the friction force between the tool and chips increases. As a result, the chip-curling radius is the highest during dry-cutting with ordinary cutting tools, making it easier for chips to wrap around the workpiece and tool, increasing chip removal resistance and leading to a sharp increase in the cutting force; this can easily result in scratches on the machined surface and even cause tool breakage. The chips generated using the textured cutting tools are spiral shaped, and the chips produced using the T9 cutting tool in Figure 7j have smaller and more uniform curling. Compared to the NTs, the textured tools produce more curled chips. This is because when chips flow through the front corner surface of the textured tools, the friction between the tool and chips is low. The friction in the contact area is small, which can reduce the curling radius of the chips and help guide them to be discharged along the texture direction. When smooth chip removal occurs, tool wear is reduced, the force is stable, and the cutting force is relatively small.

## 4. Conclusions

Compared to previous studies, this study innovatively designed a specific combination of microgroove parameters and used a femtosecond laser to process and manufacture the front cutting surface. Subsequently, a titanium alloy (Ti6Al4V) was cut under dry cutting conditions, and the influence of microgroove parameters on the performance of the tool cutting the titanium alloy was investigated through experiments. The research results can be applied to the actual processing of Ti-6Al4 V, which can aid tool design, selection, and microtexture parameter optimization. There are some limitations of this paper. Only in a dry-cutting scene was the quantitative experimental study of cutting parameters was carried out for a single material. Future work should conduct in-depth research on other alloy materials under various processing conditions and under different cutting parameter settings. The main findings and conclusions of this study are as follows:

(1) A study was conducted on microgrooved cutting tools with nine different designs, and the optimal parameter combination was found in the T9-textured cutting tool. When the groove diameter was set to 80 μm, the groove depth was 60 μm, the groove spacing was 80 μm, and the distance between the groove and the tool tip was 120 m, the tool achieved the best cutting performance (lowest cutting force, minimal wear, and optimal chip morphology).

(2) Compared to nontextured tools, textured tools with different parameter combinations reduced the cutting force. The best effect was achieved with the T9-textured tool, which reduced the cutting force by 13.9%.

(3) Compared to nontextured tools, the degree of wear of the textured cutting tools decreased (wear assessment based on SEM visual observation), with the T9-textured tool showing the most significant wear reduction and more uniform chip curling.

## Figures and Tables

**Table 1:** Parameter levels for four texture characteristics.

Parameter (μm)	Level		
	L1	L2	L3
Texture diameter	40	60	80
Depth	20	40	60
Pitch	60	80	100
Edge distance	120	150	180

**Table 2:** Taguchi L9 orthogonal design for nine texture combinations.

Experiment Tool Specimen	Diameter (μm)	Depth (μm)	Pitch (μm)	Distance (μm)
NT	0	0	0	0
T1	40	20	60	120
T2	40	40	80	150
T3	40	60	100	180
T4	60	20	80	180
T5	60	40	100	120
T6	60	60	60	150
T7	80	20	100	150
T8	80	40	60	180
T9	80	60	80	120

**Table 3:** ANOVA results for cutting force.

Parameters	SS	DOF	F	Contribution (%)
Diameter	86.222	2	1.995	49.89
Depth	20.222	2	0.468	11.65
Pitch	6.889	2	0.159	3.99
Distance	59.556	2	1.378	34.47
Total	172.889	8	___	100

## Data Availability

The original contributions presented in this study are included in the article. Further inquiries can be directed to the corresponding authors.
